# Physiological Responses to Multiple Low-Doses of *Bacillus anthracis* Spores in the Rabbit Model of Inhalation Anthrax

**DOI:** 10.3390/pathogens9110877

**Published:** 2020-10-24

**Authors:** Sarah C. Taft, Tonya L. Nichols, Stephanie A. Hines, Roy E. Barnewall, Gregory V. Stark, Jason E. Comer

**Affiliations:** 1 U.S. Environmental Protection Agency, National Homeland Security Research Center, Cincinnati, OH 45224, USA; Nichols.tonya@epa.gov; 2 Formerly of Battelle Memorial Institute, Columbus, OH 43201, USA; sj.hines@hotmail.com (S.A.H.); starkg72@gmail.com (G.V.S.); 3 Battelle Memorial Institute, Columbus, OH 43201, USA; barnewallr@battelle.org; 4 Institutional Office of Regulated Nonclinical Studies, University of Texas Medical Branch at Galveston, Galveston, TX 77555, USA; jscomer@utmb.edu; 5 Department of Microbiology and Immunology, University of Texas Medical Branch at Galveston, Galveston, TX 77555, USA; 6 Sealy Institute for Vaccine Sciences, University of Texas Medical Branch at Galveston, Galveston, TX 77555, USA; 7 Center for Biodefense and Emerging Infectious Diseases, University of Texas Medical Branch at Galveston, Galveston, TX 77555, USA

**Keywords:** *Bacillus anthracis*, anthrax, low-dose, multiple dose, dose-response, physiological response, pathology

## Abstract

*Bacillus anthracis* spores that are re-aerosolized from surface deposits after initial contamination present significant health risks for personnel involved in decontamination. To model repeated exposure to low dose *B. anthracis* spores, three groups of seven rabbits were challenged with multiple low-doses of *B. anthracis* spores 5 days a week for 3 weeks. Mortality, body temperature, heart and respiration rates, hematology, C-reactive protein, bacteremia, and serum protective antigen were monitored for 21 days post-exposure after the last of multiple doses. All rabbits exposed to a mean daily dose of 2.91 × 10^2^ colony forming units (CFU) survived and showed minimal physiological changes attributable to exposure. One of seven rabbits receiving a mean daily dose of 1.22 × 10^3^ CFU died and four of seven receiving a mean daily dose of 1.17 × 10^4^ CFU died. The LD_50_ was calculated to be 8.1 × 10^3^ CFU of accumulated dose. Rabbits that succumbed to the higher dose exhibited bacteremia and increases above baseline in heart rate, respiration rate, and body temperature. Two rabbits in the mean daily dose group of 1.17 × 10^4^ CFU exhibited clinical signs of inhalation anthrax yet survived. This study provides a description of lethality, pathophysiology, and pathology in a controlled multiple low-dose inhalation exposure study of *B. anthracis* in the rabbit model. The data suggest that the accumulated dose is important in survival outcome and that a subset of rabbits may show clinical signs of disease but fully recover without therapeutic intervention

## 1. Introduction

The release of *Bacillus anthracis* spores could result in significant contamination in both indoor and outdoor environments. Spores that are re-aerosolized from surface deposits prior or subsequent to decontamination may result in daily low-dose exposures [1]. Given the potential for severe illness and lethality from low-level inhalation exposure to *B. anthracis* spores [2], an assessment of the human health hazard must be performed to ensure safe re-use of these areas. To better characterize the hazard posed by single low-doses of *B. anthracis* Ames strain spores, Taft et al. [3] evaluated the physiological, pathological, and lethality responses of the rabbit following single inhaled low-doses. Here, we present the results of an independent follow-on study performed to evaluate multiple inhaled low-doses of *B. anthracis* Ames strain spores in the rabbit model. 

Currently, there is no consensus on the dose-relationship or the most appropriate calculation of dose to perform dose-response analysis on the human health effects of single or multiple exposures to *B. anthracis* spores [4,5]. Published data describing multiple dose exposures, defined as those that incorporate more than one dosing event at the same or differing doses, are rare for pathogens generally and *B. anthracis* specifically. Two published studies report the response of the nonhuman primate (NHP) for multiple dose inhalation exposures to *B. anthracis* spores [6,7]. Albrink and Goodlow [6] reported the results of a controlled exposure multiple dose challenge study for the NHP (*n* = 2) with a 6-week period between doses. Brachman et al. [7] described the multiple dose exposures of cynomolgus monkeys to uncontrolled *B. anthracis* levels from mill operations over ranges of days to months. However, the raw data remain unpublished and inhalation rates must be estimated from the figures in the manuscript [8]. 

Given the lack of multiple dose studies for low-dose exposures describing lethality, pathophysiology, and pathology for the rabbit, further study is needed to develop the necessary body of data for the generation of dose-response relationships or assessment of remediation for *B. anthracis* events. To continue efforts to fill the identified data gaps for inhalation anthrax risk assessment, this study was performed to evaluate physiological, pathological, and lethality responses following multiple low-dose exposure of *B. anthracis* Ames strain spores in the rabbit model. The data presented here were originally reported to the U.S. Environmental Protection Agency [9] and used to model dose responses in rabbits [10,11]. With this article, the authors intend to provide the data to the broader research community and build on recently published data involving a single low-dose exposure [3]. 

## 2. Materials and Methods

All of the following select agent work was conducted at the Battelle Biomedical Research Center (West Jefferson, OH, USA) in a Biosafety Level-3/Animal Biosafety Level-3 laboratory registered with the Centers for Disease Control and Prevention (CDC) and inspected by the U.S. Department of Defense and the U.S. Department of Agriculture. The animal procedures and protocols described herein were pre-approved by Battelle’s Institutional Animal Care and Use Committee (IACUC) and the Department of Defense’s Animal Care and Use Review Office (ACURO). The research was conducted in compliance with the federal Animal Welfare Act and followed the principles in the Guide for the Care and Use of Laboratory Animals from the National Research Council [12]. The institution where the research was conducted is fully accredited by the Association for the Assessment and Accreditation of Laboratory Animal Care International (AAALAC). 

All work was conducted following an approved Test/Quality Assurance Plan to ensure that sufficient quality objectives and performance criteria were met for determination of the adequacy of data generated during the study. Further detail on the quality assurance process can be found in the project report [9].

### 2.1. Bacillus anthracis Ames Spores and Aerosol Challenges

The *B. anthracis* Ames strain spores were prepared and characterized as previously described in Barnewall et al. [1]. The spore suspensions contained 0% vegetative cells and 0.34% debris when examined with phase contrast microscopy (Leica Microsystems, Wetzlar, Germany), and 99.28% of the spores were refractile. Spores used in the negative control group were inactivated with 4 × 10^6^ rad doses of gamma irradiation in a CDC laboratory using a method described by Dauphin et al. [13]. Inactivation was verified via no growth on culture plates. All rabbits were challenged with aerosolized *B. anthracis* Ames spores as previously described [1].

Based on the results of the previous single dose study [3], it was decided to challenge with repeated targeted doses of 1.0 × 10^2^, 1.0 × 10^3^ and 1.0 × 10^4^. Three groups of seven New Zealand White (NZW, *Oryctolagus cuniculus*) male rabbits were challenged by receiving mean inhaled daily doses ranging from 2.91 × 10^2^ colony forming units (CFU) to 1.17 × 10^4^ CFU of aerosolized *B. anthracis* Ames spores (Table 1). A group of five animals was exposed to daily doses of 1.0 × 10^4^ gamma irradiated spores and served as negative controls at the highest daily dose of live spores. Group doses were calculated and reported as the arithmetic mean of the individual mean inhaled doses, with the individual mean inhaled dose equal to the total dose divided by the number of challenge days. The challenges occurred Monday through Friday for three consecutive weeks; there were no weekend challenges.

Data were collected throughout the study, defined as 7 days prior to the first challenge and for 21 days after the last challenge day. 

### 2.2. Telemetry Analysis

Body temperature, respiration rate, heart rate, and activity were recorded continuously throughout the study using implanted D70-PCT transmitters (Data Sciences International, St. Paul, MN, USA as described for the single dose study in Taft et al. [3]. 

### 2.3. Necropsy and Histopathology

Animals that succumbed to challenge or were found moribund and euthanized underwent gross necropsy. Surviving animals were euthanized and necropsied on Study Day 39. Lungs and any gross lesions were collected and examined for histopathology as described in Taft et al. [3].

### 2.4. Hematology and C-Reactive Protein 

Blood samples were collected via surgically-implanted vascular access ports (VAP) (Covance) on Study Days –3, 2, 4, 9, 11, 16, 18, 23, 25, 30, 32, and 37, and on the day of death (i.e., terminal sample). Hematological analysis was performed using the Advia 120 Hematology Analyzer (Siemens Healthcare Diagnostics, Newark, DE, USA) and C-reactive protein (CRP) levels were measured in plasma using the Advia^®^ 1200 chemistry analyzer (Siemens Healthcare Diagnostics, Newark, DE, USA). 

### 2.5. Bacteremia Analysis

Bacteremia was assessed by quantitative culture on blood agar plates as described in Taft et al. [3]. Bacteremia (quantitative culture) was assessed on all blood sample Study Days as collected material allowed. 

### 2.6. Toxemia Analysis

The circulating PA enzyme linked immunosorbent assay (ELISA) was performed as described in Comer et al. [14]. The limit of detection (LOD) for this assay was 2.0 ng/mL and the lower limit of quantification (LLOQ) was 4.9 ng/mL.

### 2.7. Serology Analysis

To determine if the rabbits seroconverted following challenge, serum samples were analyzed by ELISA and high-throughput toxin neutralization assay (htp-TNA) as described in Ionin et al. [15]. Serum samples were analyzed from blood samples collected via surgically-implanted VAP (Covance) on Study Days –3, 4, 11, 18, 25, 32, and 39, and a terminal sample.

### 2.8. Statistical Analysis

Statistical analyses were performed using R (The R Foundation for Statistical Computing, version 2.9.2, Vienna, Austria). 

Estimates and exact Clopper–Pearson 95% binomial confidence intervals for the proportion of surviving animals were calculated for each group. An overall two-sided Fisher’s exact test was performed to determine if the proportions of surviving animals were significantly different among the groups. If the overall Fisher’s exact test was significant, then pairwise two-sided Fisher’s exact tests were performed to determine which pairs of groups were significantly different from each other. A Bonferroni–Holm adjustment was made to maintain an overall 0.05 level of significance for the multiple pairwise comparisons. A logistic regression model was fitted to the survival data as a function of the base 10 logarithm of the estimated inhaled dose to determine the effect of dose on lethality. The LD_50_ was estimated from the logistic regression model, along with 95% Fieller’s confidence intervals.

The mean telemetry value was computed for every 15-min clock time reported in military time units (0000, 0015, … 2400) at baseline. Each observation was then baseline adjusted according to the associated clock time and 6-hour averages were computed from the baseline adjusted values using the following intervals: 0000–0600 (inclusive), 0600–1200 (inclusive), 1200–1800 (inclusive), and 1800–2400 (inclusive). In order to determine if the baseline adjusted telemetry results were significantly different among the groups, analysis of variance (ANOVA) models were fit to the baseline-adjusted 6-hour average telemetry values with an effect for group at each study time. Least square mean estimates from the ANOVA models were calculated and approximate t-tests were performed to determine if, for each group, there was a significant shift in the telemetry values between baseline and each study time, after adjusting for the clock time. Additionally, Tukey’s multiple comparisons procedure was performed to determine which pairs of groups had mean baseline-adjusted telemetry values that were significantly different from each other. Under the Tukey procedure, the set of all comparisons within each parameter and study time combination are made at a joint 95% confidence level.

The standard deviation of each 6-hour average at baseline was calculated and used to form the upper and lower limits for indications of abnormality for telemetry measures of heart rate, respiration rate, and body temperature. The upper limit was defined to be three standard deviations above zero, while the lower limit was defined to be three standard deviations below zero. An animal was found to be abnormal if two consecutive baseline-adjusted 6-hour averages were outside the upper or lower limits following challenge. The time of onset for abnormality was defined as the time associated with the second abnormal value during the first occurrence of two consecutive abnormal values following challenge. The end of abnormality was defined as the time associated with the last abnormal value during the last occurrence of two consecutive abnormal values following challenge. Therefore, the duration of abnormality was defined as the difference between the time associated with the end of abnormality and the time associated with the onset of abnormality. Fisher’s exact tests were performed to test for significant differences in the proportion of animals abnormal among the groups. Log-rank tests were performed to test for significant differences in time to abnormality and duration of abnormality among the groups.

The PA and quantitative bacteremia data were log-transformed for the statistical analyses. The assumption of normality was deemed reasonable for the log-transformed values. All PA measurements less than the LOD were replaced with one-half of the LOD. All quantitative bacteremia measurements less than the LOD (reported as zero CFU/mL) were replaced with one-half of the LOD. Furthermore, if an observation was positive for *B. anthracis* but less than the LOQ, then it was replaced with one-half of the LOQ. The replacement of measurements less than the LOD or LOQ with one-half of its value was conducted only for the statistical analysis; reported values unrelated to the statistical analysis were not altered. T-tests were performed to determine if the geometric mean was significantly greater than the LOD for each parameter, group, and study day. For each hematology parameter, ANOVA and Tukey’s multiple comparisons procedures were performed at each study time to determine if the mean shifts from baseline were significantly different among the groups. The ANOVA models were also used to test if parameter values were significantly different from baseline for each group and time point.

## 3. Results

### 3.1. Mortality

Rabbits were exposed to low-doses of *B. anthracis* spores over 15 challenge days with group mean daily challenge doses that ranged from 2.91 × 10^2^ to 1.17 × 10^4^ CFU (Table 1). All rabbits exposed to the irradiated spores and all in the 2.91 × 10^2^ CFU daily mean dose group survived until the end of the study. Rabbit 2, one of the seven animals in the 1.22 × 10^3^ CFU daily dose group, died 17.9 days after the first exposure. This animal received 14 of the 15 challenge doses and received an accumulated challenge dose of 1.86 × 10^4^ CFU over the course of the study. Four of the seven rabbits in the 1.77 × 10^4^ CFU mean daily dose group succumbed to disease with a mean time to death of 14.80 ± 4.28 days. The mean accumulated dose of the four animals that died was 1.11 × 10^5^ CFU. Three animals died prior to the administration of the full 15 challenge doses with times to death of 10.9, 12.7, and 14.7 days (Table 1 and Supplementary Figure S1).

Of the nine animals that received an accumulated challenge dose in the 10^4^ level of magnitude, three of nine died from inhalation anthrax. Of the five animals that received an accumulated challenge dose in the 10^5^ level of magnitude, two of five died from inhalation anthrax (Table 1). The LD_50_ value calculated using a logistic regression model and an accumulated inhaled dose metric was 8.1 × 10^3^ CFU, with a 95% Fieller’s confidence interval of 2.3 × 10^3^ CFU to 3.6 × 10^7^ CFU.

### 3.2. Necropsy and Histopathology

Gross lesions were consistent with inhalation anthrax in rabbits [16] and included discoloration of the lungs, foci in the appendix, “accumulation” in the cecum, and/or enlargement of a mediastinal lymph node; these lesions were identified in Rabbit 12 (1.22 × 10^3^ CFU mean daily dose animal), Rabbit 6 (1.17 × 10^4^ CFU mean daily dose group), Rabbit 33 (1.17 × 10^4^ CFU mean daily dose group), and Rabbit 27 (1.17 × 10^4^ CFU mean daily dose group). Rabbit 12 was also observed as having a lesion on its left hind limb. This lesion was most probably due to an injury that occurred outside of study activities. Pathology findings for individual rabbits are provided in Supplemental Material (Supplementary Table S1). Microscopic findings consistent with anthrax as described by Zaucha et al. [16] were present in tissues from all rabbits that died from inhalation anthrax in the 1.22 × 10^3^ CFU mean daily dose and 1.17 × 10^4^ CFU mean daily dose groups. Lesions typical of anthrax in this study included suppurative inflammation, necrosis, lymphocyte necrosis/depletion, hemorrhage, edema, and/or large rod-shaped bacteria (bacilli) in the lungs, cecum, appendix, and mediastinal lymph nodes. 

Lesions in the lung attributed to *B. anthracis* were primarily interstitial and consisted of minimal to mild suppurative interstitial inflammation with interstitial and/or intravascular bacteria. Rabbit 38, in the 1.17 × 10^4^ CFU mean daily dose group, that survived inhalation anthrax exhibited some gross pathology in the lung (i.e., discoloration, pale) and perivascular eosinophils at sacrifice. Gross lesions in the lungs in Rabbit 38 were correlated with multiple foreign body granulomas and pyrogranulomas. However, Rabbit 21, also in the 1.17 × 10^4^ CFU mean daily dose group, survived inhalation anthrax with no remarkable pathology reported at the end of study. Other rabbits in the same group that died from inhalation anthrax exhibited mild suppurative inflammation and/or minimal perivascular eosinophils, with some lesions identified in other organs or tissues (e.g., cecum, lymph nodes, or appendix). Rabbit 39, from the same dose group that did not exhibit signs of inhalation anthrax, was reported to have minimal foreign bodies and multinucleated giant cells in the lung, with no other lesions noted. 

In the mean daily 2.91 × 10^2^ CFU and 1.22 × 10^3^ CFU dose groups, animals challenged with live spores but without evidence of anthrax infection presented with either unremarkable microscopic findings in the lung or mild to minimal perivascular eosinophils and mild multinucleated giant cells in the lung.

### 3.3. Physiological Responses

#### 3.3.1. Telemetry

**Figure 1:**
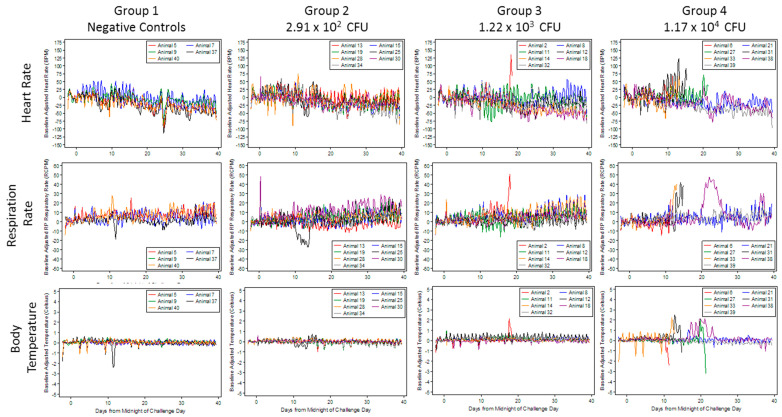
Telemetry monitoring of rabbits receiving multiple low-doses of *B. anthracis* spores. Three groups of seven rabbits were exposed to mean daily doses ranging from 2.91 × 10^2^ (± 3.88 × 10^2^) CFU to 1.17 × 10^4^ (± 4.64 × 10^3^) CFU. A group of five animals were exposed to 1.00 × 10^4^ irradiated spores and served as negative controls. The challenges occurred Monday through Friday for 3 consecutive weeks. The rabbits were surgically implanted with telemetry units (D70-PCT transmitters, Data Sciences International, St. Paul, MN, USA) prior to being placed on study. Each D70-PCT transmitter contained one pressure lead and one biopotential lead. Body temperature, electrocardiogram activity, and cardiovascular function were monitored for 30 s every 15 min for 7 days pre-challenge (baseline) through the end of the study.

Figure 1 illustrates the changes in telemetry parameters on an individual animal basis through the course of the study. Intermittent decreases during blood collection times for heart rate, respiration rate, and body temperature are attributable to the use of acepromazine. By Study Day 1 at 1200–1800, all groups had experienced significant increases from baseline heart rate. These significant increases continued intermittently for all groups until Study Day 5 at 0600–1200. This increase in heart rates across groups may be associated with increased study activity in the animal room (i.e., study-related stress). All rabbits succumbing to disease presented with an increase in heart rate above baseline, with some reaching 125 beats per minute above baseline readings (Figure 1). 

By Study Day 1 at 1200–1800, all groups had also experienced a significant increase from baseline in respiration rates. As with the heart rate data, the increases in respiration were most probably due to study-related stress. All rabbits that died on study presented with an increase in respiration rate above baseline immediately prior to death. This was most pronounced in Rabbit 2 (1.22 × 10^3^ mean daily dose group) and Rabbits 6 and 31 (1.17 × 10^4^ mean daily dose group) where respiration rates increased to over 40 respiratory cycles per minute prior to death. Rabbit 38 (1.17 × 10^4^ mean daily dose group) showed an increase in respiration rate above baseline from approximately Study Days 21 to 26 but returned to the baseline rate and lived to the end of the study.

Except Rabbit 6 (1.17 × 10^4^ CFU mean daily dose group), all animals that succumbed to infection showed an increase from baseline body temperature prior to death. These increases were approximately 2 °C above baseline. Rabbit 38 (1.17 × 10^4^ CFU mean daily dose group) also had an increase in body temperature from baseline from Study Days 18 through 24, which corresponded with the time that the animal was also exhibiting an increase in respiration rate above baseline. Rabbit 21 (1.17 × 10^4^ mean daily dose group) presented with a moderate increase in body temperature above baseline on Study Day 17 but all other tested parameters tested did not depart from baseline. There were no significant differences between the dose groups (i.e., no dose-dependency in response) for any of the telemetry parameters with regard to the proportion of animals that became abnormal, time to abnormality, and duration of abnormality.

**Figure 2:**
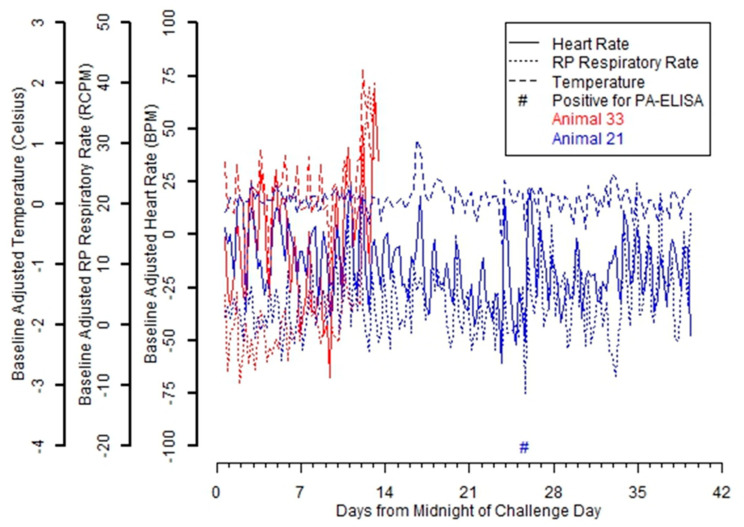
Individual telemetry, bacteremia, and toxemia results from the multiple low-dose study for Rabbit 33 that died from inhalation anthrax and Rabbit 21 that survived the challenge. From the 1.17 × 10^4^ CFU multiple low-dose group, individual results from a rabbit that died from inhalation anthrax (Rabbit 33) are plotted with individual results for a representative rabbit survivor (Rabbit 21) of the challenge that developed an inhalation anthrax infection but did not die.

Notable differences were observed when comparing individual telemetry results for a representative animal that was infected with inhalation anthrax but did not succumb to the infection (Rabbit 21) versus one that was infected and died from inhalation anthrax (Rabbit 33) (Figure 2). Rabbit 33 exhibited spikes in baseline-adjusted heart rate, temperature, and respiratory rate near Study Day 12 (Figure 2). The time to abnormality for heart rate, respiratory rate, and temperature was 13.375 days, 12.375 days, and 13.125 days, respectively (data not shown). Bacteremia was not detected on Study Days 9 or 11 when assayed, and the animal was bacteremic for the terminal sample at death in the latter half of Study Day 12.

Rabbit 21 developed an inhalation anthrax infection, as evidenced by the positive detection of toxemia, but did not succumb (Figure 2). The large spikes in baseline adjusted measures identified in Rabbit 33 were not observed in Rabbit 21 though occasional variability was noted (e.g., near Day 17), especially prior to seroconversion. However, abnormality as measured relative to the baseline adjusted measures was not observed and bacteremia was not detected in this animal at any time in the study.

#### 3.3.2. Hematology and C-Reactive Protein

**Figure 3:**
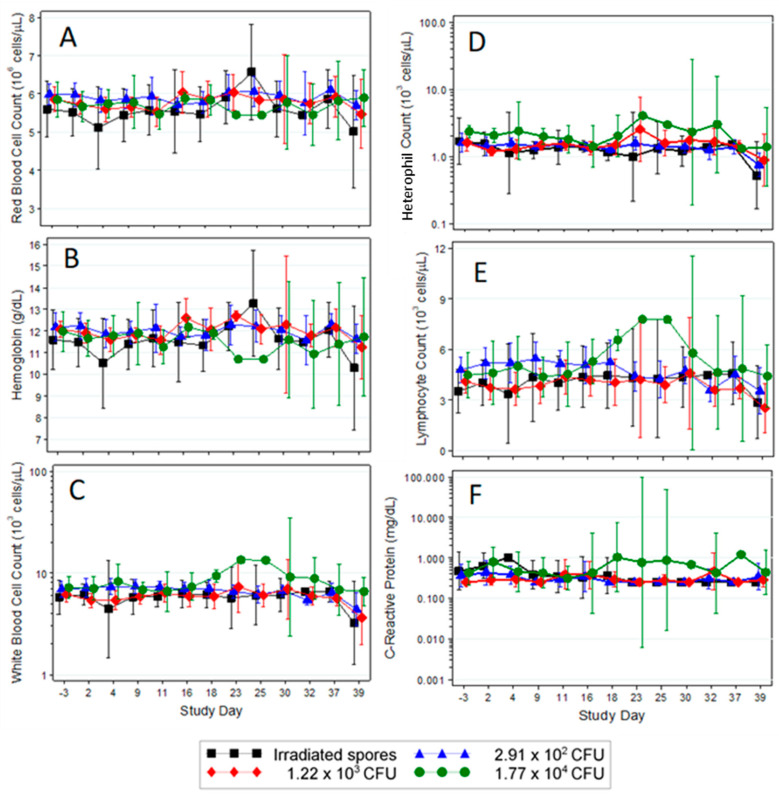
Changes in hematology and C-reactive protein levels following multiple low-doses of *B. anthracis* spores. On Study Days –3, 2, 4, 9, 11, 16, 18, 23, 25, 30, 32, and 37, blood samples were collected and hematological analysis was performed using the Advia 120 Hematology Analyzer [Siemens Healthcare Diagnostics; red blood cell counts (**A**), hemoglobin concentration (**B**), white blood cell counts (**C**), neutrophil counts (**D**), lymphocyte counts (**E**)]. C-reactive protein (CRP; Panel **F**) levels were measured in plasma using the Advia^®^ 1200 chemistry analyzer (Siemens Healthcare Diagnostics, Newark, DE, USA). Points represent the means for individual groups with error bars showing the 95% confidence intervals. Please note, the x axis is presented as categorical not numerical.

Significant changes were identified across several hematology parameters. There were significant group effects in the red blood cell (RBC) counts (Figure 3A). On Study Day 23, the mean decrease from baseline in the 1.17 × 10^4^ CFU mean daily dose group was significantly different than the mean increase from baseline in the 1.22 × 10^3^ CFU mean daily dose group (*p* = 0.0489, Tukey’s test). On Study Day 25, the mean decrease from baseline in the 1.17 × 10^4^ CFU mean daily dose group was significantly different than the mean increases from baseline in the irradiated spore group (*p* = 0.0023, Tukey’s test) and the 2.91 × 10^2^ CFU mean daily dose group (*p* = 0.0229, Tukey’s test). While the changes were statistically significant, their relevance is not apparent as all rabbits remained in or very close to the normal range of 4.20–6.70 × 10^6^ RBCs/µL. Individual hematology results are provided in Supplementary Table S2.

There were also significant changes in the hemoglobin (HGB) concentrations in the blood (Figure 3B). The decrease from baseline in the irradiated spore group on Study Day 4 was significant (*p* < 0.05, approximate t-test). Like the RBC counts, the HGB concentrations, while low, were within or very near the normal range (9.5–14.5 g/dL; Figure 3B) and statistical differences were not likely to be clinically relevant. 

The mammalian host responds to extracellular bacterial infection by increased hematopoiesis and heterophilia. To determine if the rabbits responded to the multiple exposures of *B. anthracis*, complete white blood cell (WBC) counts and differentials were performed. There were no significant shifts as a proportion of baseline and no significant differences between the groups on any post-challenge study day for WBC, neutrophil, or lymphocyte counts. While not statistically significant, Figure 3C–E shows that surviving animals challenged with a mean daily dose of 1.17 × 10^4^ CFU had higher circulating levels of these cells than the other groups.

Rabbit 38 from the 1.17 × 10^4^ CFU daily dose group presented with increases above baseline in heart rate, respiration rate, and body temperature, and lived to the end of the study. Rabbit 38 also exhibited an increase in WBCs well above the normal range of 2.90–8.10 × 10^3^ WBCs/µL. In fact, the WBC count reached 2.33 × 10^4^ cells/µL on Study Day 23 but decreased back into the normal range by the end of the study (data not shown). Rabbit 21, a survivor from the same dose group, also presented with increases above baseline in body temperature and toxemia. However, the WBC data for Rabbit 21 are difficult to interpret because the initial WBC measurement at Day 3 was slightly above the normal range (8.37 × 10^3^ WBC/µL), decreased to the normal range, peaked on Study Day 18 at 8.62 × 10^3^ WBC/µL, and then decreased to within the normal range for all successive measurements for which sufficient sample volumes were obtained (Supplementary Table S2).

There was a significant increase in CRP when compared to baseline in the 1.17 × 10^4^ CFU daily dose group on Study Day 2 (*p* > 0.05, approximate *t*-test) (Figure 3F, Supplementary Table S2). There were no significant differences among the groups on any post-challenge study day. The normal levels range from 0.25 to 0.29 mg/dL [17,18]. The increase in CRP levels did not correspond with morbidity or mortality and in most cases could have resulted from the stress of study activity. Rabbit 38 showed the highest levels of CRP between Study Days 18 and 25, with the maximum measurement of 7.42 mg/dL on Study Day 23. Individual rabbit levels of CRP are provided in Supplementary Table S3.

#### 3.3.3. Bacteremia

All animals that received irradiated spores or a daily mean dose of 2.91 × 10^2^ CFU were negative for *B. anthracis* bacteremia by culture on all study days. The terminal sample from the 1.22 × 10^3^ CFU mean daily dose animal (Rabbit 2) that succumbed to infection showed a bacterial load in the blood of 3.87 × 10^5^ CFU/mL and earlier cultures were negative. The rest of the animals in this dose group were not observed to be bacteremic. The terminal samples of three out of the four rabbits that died in 1.17 × 10^4^ CFU mean daily dose group were positive for bacteremia. Rabbits 33, 27, and 31 had terminal bacteremia levels of 4.13 × 10^5^ CFU/mL, 2.60 × 10^3^ CFU/mL, and 4.00 × 10^1^ CFU/mL (<LLOQ), respectively. One of the non-survivors of this group (Rabbit 6) was not observed to be bacteremic by culture and was found dead 10.9 days after the first challenge. Rabbit 38 became bacteremic on Study Day 18 (1.80 × 10^2^ CFU/mL) (<limit of quantification [LOQ]), which resolved by the next blood collection time (Study Day 23). The other two rabbits that survived to the end of the study (Rabbits 39 and 21) were not observed to be bacteremic. 

#### 3.3.4. Toxemia

Toxemia was assessed over the course of the study via a PA ELISA test, which measured circulating levels of PA. All rabbits that received irradiated spores or a mean daily dose of 2.91 × 10^2^ CFU were below the LOD (4.9 ng/mL) at all time points assayed. The 1.22 × 10^3^ CFU mean daily dose group animal (Rabbit 2) that died on Study Day 17 had 159 ng/mL of PA detected in the terminal blood sample; all other rabbits in this group were below the LOD throughout the study. Only one of the four rabbits (Rabbit 27) in the 1.17 × 10^4^ CFU mean daily dose group that succumbed to disease had detectable levels of PA in the terminal sample (65,300 ng/mL PA). Two survivors (Rabbit 38 and Rabbit 21) from this group had detectable levels of PA. Rabbit 38 had 7.70 and 6.28 ng/mL PA on Study Days 18 and 23, respectively. However, the toxemia resolved by Study Day 30. Rabbit 21 had 4.97 ng/mL PA on Day 25; all other blood samples were below the LOD. 

#### 3.3.5. Serology 

Serum samples taken on Study Days –3, 4, 11, 18, 25, 32, and 39 were analyzed via TNA and anti-PA IgG ELISA to determine if the rabbits developed a humoral response to the repeated *B. anthracis* exposures. Only Rabbit 38 (1.17 × 10^4^ CFU mean daily dose group) had detectable levels of antibodies by either method. No other animal had detectable levels of antibodies using either method of measurement. The htp-TNA was used to determine the effective dose 50% (ED_50_) and neutralization factor 50% (NF_50_) of sera able to neutralize lethal toxin. The ED_50_ values for Rabbit 38 on Study Days 25, 32, and 39 were 5858, 12789 and 7250, respectively. The NF_50_ on these study days were 12.71, 26.44 and 14.82, respectively. The immunoglobulin G (IgG) ELISA results showed that Rabbit 38 had 1636.02, 2190.85 and 1728.47 µg/mL of circulating anti-PA IgG on Study Days 25, 32 and 39, respectively. 

## 4. Discussion 

To model repeated low-level exposure to *B. anthracis* spores following re-aerosolization from contaminated surfaces, we challenged NZW rabbits with multiple low doses of *B. anthraces* spores. NZW rabbits have long been an established model of inhalational anthrax and recapitulates many of the characteristics of the human disease [16,19]. Available multiple dose data for *B. anthracis* exposure were unavailable for the rabbit and scarce for other animal models. Two studies report results of multiple dose exposures in the NHP [6,7]. These studies have limitations regarding the number of animal subjects, availability of raw dose data, and comparability of collected data that preclude the use of statistical comparisons with the currently reported multiple-dose study. 

Taft et al. [3] reported pathophysiology, pathology, and lethality results for a single inhaled low-dose exposure of *B. anthracis* spores in the rabbit. Here, the data regarding the signal dose study was expanded by exposing rabbits to multiple low doses. All of the rabbits exposed to 15 daily doses with a mean of 2.91 × 10^2^ CFU lived to the end of this multiple dose study and showed minimal physiological changes due to the exposures. Only one rabbit (Rabbit 2) in the 1.22 × 10^3^ CFU mean daily dose group died of anthrax on Study Day 17.9. Rabbits surviving after an anthrax challenge usually seroconvert by Day 7 with titers increasing to Day 14 post-challenge [20,21]. Rabbit 2 never seroconverted, suggesting an infection was not established until completion of the first 2 weeks of challenges.

In the highest multiple dose group, four of seven rabbits died. The rabbits that died from inhalation anthrax after receiving a 10^4^ CFU level of magnitude accumulated dose presented with increases above baseline in heart rate and respiration rate. An increase above baseline in body temperature was reported in all rabbits that died from inhalation anthrax after receiving a 10^4^ CFU level of magnitude accumulated dose, except for one rabbit (Rabbit 6).

Bacteremia was not detected until the terminal sample in four of the five non-surviving rabbits and was not detected in the terminal sample of one non-surviving rabbit (Rabbit 6). Bacteremia was detected, but below the countable range, in Rabbit 38 that survived inhalation anthrax. One potential explanation for the lack of positive bacteremic measurements may be the presence of measurable bacteremia occurred only during the gap days in the blood collection days (i.e., Study Days 3, 5–8, 10, 12–15, 17 and 19–22). Furthermore, most rabbits that succumb to inhalation anthrax from single high-dose exposures have detectable PA levels in the late stage blood samples [14,22,23,24]. It has been established that inhalation anthrax is a biphasic or triphasic disease with a brief remission of bacteremia and toxemia in NHPs [25,26,27]. It is possible that inhalational anthrax in the rabbit follows the same course wherein these animals had circulating bacteria and PA during the course of the infection, but the times of bacteremia and toxemia did not coincide with the blood collections. 

The data generated in the single dose study suggested an “all-or-none” outcome to disease [3]. The establishment of infection, as marked by bacteremia and physiological changes, resulted in disease progression to a fulminant state and ultimately death. A similar “all-or-none” aspect to fulminant anthrax disease presentation for single dose exposure was previously reported by Zaucha et al. [16]. Here it is reported that two animals (Rabbits 38 and 21 in the highest mean daily dose group (1.17 × 10^4^ CFU mean daily dose group) showed physiological signs of infection but lived to the end of the study. Rabbit 38 presented with clinical signs of inhalation anthrax (i.e., increases above baseline in heart rate, respiration rate, and body temperature), bacteremia, and toxemia during the study. The heterophilic response in Rabbit 38 was more robust than that of the other animals, and Rabbit 38 seroconverted on Study Day 25. Another survivor in this group, Rabbit 21, presented with a fever, neutrophilia, and toxemia, but seroconversion was not observed by ELISA or htp-TNA.

The two rabbits that survived inhalation anthrax either exhibited low level transient bacteremia (Rabbit 38), or possibly bacteremia levels that were below the detection limit given the presence of toxemia (Rabbit 21). Survival of inhalation anthrax with observed. Low-level transient bacteremia is also reported for the NHP after a two-dose challenge [6] and a single high-dose challenge [28,29]. This may indicate that the development of bacteremia and toxemia is not a uniform predictor of lethality, but that the progression of physiological signs indicative of severe systemic illness is necessary for lethality. 

NZW rabbits succumb to a lethal aerosol dose of *B. anthracis* spores within 2 to 3 days. Here, we show that multiple sub lethal doses can accumulate and ultimately cause death. *B. anthracis* spores undergo asynchronous germination thus allowing the accumulation of spores and vegetative bacteria to reach lethal levels. 

In summary, this study reported lethality, pathophysiology, and pathology results from multiple low-dose inhalation exposures of aerosolized *B. anthracis* spores in the rabbit animal model. The data suggest inhalational anthrax is not 100% lethal in rabbits, even after signs of disease are present. Furthermore, this study provided evidence that low doses of *B. anthracis* spores can accumulate in exposed individuals and ultimately cause death. The survival results of the single challenge study [3] and multiple challenge data presented here have been used in dose-response computer modeling by our group and others [10,11]. The next step is to use the physiological data presented here to predict non-lethal responses to repeated exposure to low doses of *B. anthracis* spores in humans.

## 5. Acknowledgements

The authors gratefully acknowledge the permission granted by the National Institutes of Health (NIH) to use their PA enzyme-linked immunosorbent assay (ELISA) and toxin neutralization assay (htp-TNA) reagents, which were developed by Battelle for use in a previous NIH study. The authors would also like thank Alex Hoffmaster and Laura Rose of the Centers for Disease Prevention and Control (CDC) for irradiating the spores used in this work. The authors are also grateful for the thoughtful and thorough reviews provided by Dr. Harry Stone (Battelle), Dr. M. Worth Calfee (U.S. EPA, National Homeland Security Research Center), and Dr. Harlal Choudhury (U.S. EPA, National Center for Environmental Assessment).

## Figures and Tables

**Table 1:** Individual Daily Challenge Dose of Survival Outcome.

Group Mean Daily Inhaled Dose, in 1.0 × 10^3^ CFU (Standard Deviation) ^a^ and Particle Size (GSD) ^b^	Rabbit ID Number	Individual Mean Daily Inhaled Dose		Number of Doses	Accumulated Dose (CFU) ^c^	Outcome, Survived or Died (Days from First Challenge Day to Death)
		Mean	Standard Deviation			
Irradiated Spores 0.81 µm (1.53)	40	0	0	15	0	Survived
	7	0	0	15	0	Survived
	5	0	0	15	0	Survived
	9	0	0	15	0	Survived
	37	0	0	15	0	Survived
0.291 (0.388) 0.79 µm (1.52)	13	3.85 × 10^2^	7.57 × 10^2^	15	5.78 × 10^3^	Survived
	34	3.17 × 10^2^	4.48 × 10^2^	15	4.76 × 10^3^	Survived
	25	2.79 × 10^2^	3.54 × 10^2^	15	4.19 × 10^3^	Survived
	15	3.17 × 10^2^	3.27 × 10^2^	15	4.76 × 10^3^	Survived
	30	2.72 × 10^2^	2.33 × 10^2^	15	4.07 × 10^3^	Survived
	28	2.34 × 10^2^	1.49 × 10^2^	15	3.51 × 10^3^	Survived
	19	2.32 × 10^2^	1.28 × 10^2^	15	3.48 × 10^3^	Survived
1.22 (0.559) 0.82 µm (1.53)	14	7.38 × 10^2^	2.99 × 10^2^	15	1.11 × 10^4^	Survived
	11	1.12 × 10^3^	5.01 × 10^2^	15	1.68 × 10^4^	Survived
	2	1.33 × 10^3^	5.95 × 10^2^	14	1.86 × 10^4^	Died (17.9)
	8	1.41 × 10^3^	6.06 × 10^2^	15	2.12 × 10^4^	Survived
	12	1.30 × 10^3^	4.90 × 10^2^	15	1.96 × 10^4^	Survived
	18	1.21 × 10^3^	5.47 × 10^2^	15	1.82 × 10^4^	Survived
	32	1.44 × 10^3^	5.92 × 10^2^	15	2.16 × 10^4^	Survived
11.7 (4.64) 0.86 µm (1.49)	6	6.41 × 10^3^	2.57 × 10^3^	9	5.77 × 10^4^	Died (10.9)
	33	9.75 × 10^3^	2.58 × 10^3^	10	9.75 × 10^4^	Died (12.7)
	27	1.06 × 10^4^	3.51 × 10^3^	14	1.59 × 10^5^	Died (20.8)
	31	1.25 × 10^4^	3.27 × 10^3^	11	1.37 × 10^5^	Died (14.8)
	39	1.44 × 10^4^	5.99 × 10^3^	15	2.16 × 10^5^	Survived
	21	1.32 × 10^4^	4.97 × 10^3^	15	1.98 × 10^5^	Survived
	38	1.27 × 10^4^	3.77 × 10^3^	15	1.91 × 10^5^	Survived

^a^ Group mean inhaled doses were calculated and represented as the arithmetic mean of the individual mean inhaled doses (CFU = colony forming units), with the individual mean inhaled doses equal to the total dose divided by the number of challenge days (15 days). ^b^ Particle size measured as mass median aerodynamic diameter (µm) with accompanying geometric standard deviation (GSD). ^c^ Accumulated defined as the total sum of individual doses for an animal.

## Supplementary Materials

The following are available online at https://www.mdpi.com/2076-0817/9/11/877/s1, Figure S1: Kaplan Meier Survival Curve, Table S1: Individual Pathology Findings, Table S2: Individual Hematology Data, Table S3: Individual CRP Results.
