# Supplementary material for: Physiological Responses to Multiple Low-Doses of Bacillus anthracis Spores in the Rabbit Model of Inhalation Anthrax
Source: Pathogens. 2020 Oct 24;9(11):877. doi: 10.3390/pathogens9110877 (PMC7693690; doi:10.3390/pathogens9110877)
Supplement: Supplementary file 1 [file pathogens-09-00877-s001.zip › Table S1 Individual Pathology Finding.docx]

Table S1. Summary of individual gross and microscopic observations

| **Group Mean Daily Inhaled Dose** | **Animal Number/ Death Status** | **Gross Findings** | **Microscopic Findings** |
| --- | --- | --- | --- |
| Irradiated Spores | 40/FS |  | Lung: Unremarkable. |
|  | 7/FS |  | Lung: Unremarkable. |
|  | 5/FS |  | Lung: Perivascular eosinophils, minimal. |
|  | 9/FS |  | Lung: Perivascular eosinophils, minimal. |
|  | 37/FS |  | Lung: Unremarkable. |
| 2.91 × 10^2^ CFU | 13/FS |  | Lung: Perivascular eosinophils, minimal. |
|  | 34/FS |  | Lung: Foreign body, mild.  Lung: Multinucleated giant cells, mild. |
|  | 25/FS |  | Lung: Unremarkable. |
|  | 15/FS |  | Lung: Perivascular eosinophils, minimal. |
|  | 30/FS |  | Lung: Unremarkable. |
|  | 28/FS |  | Lung: Perivascular eosinophils, mild. |
|  | 19/FS |  | Lung: Unremarkable. |
| 1.22 × 10^3^ CFU | 14/FS |  | Lung: Perivascular eosinophils, minimal. |
|  | 11/FS |  | Lung: Perivascular eosinophils, minimal. |
|  | 2/FD |  | Lung: Hemorrhage, minimal.  Lung: Inflammation, suppurative, minimal.  Lung: Bacteria, minimal. |
|  | 8/FS |  | Unremarkable. |
|  | 12/FS | Skin: Laceration(s), red, left hindlimb, 40 x 20 mm | Lung: Foreign body, minimal.  Lung: Multinucleated giant cells, mild.  Skin: Inflammation, necrosuppurative, marked. |
|  | 18/FS |  | Lung: Unremarkable. |
|  | 32/FS |  | Lung: Perivascular eosinophils, minimal. |

| 1.17 × 10^4^ CFU | 6/FD | Cecum: Accumulation (gas)*.* Samples of cecum, colon, jejunum, and appendix were collected to confirm lesion. | Cecum: Edema, mild.  Cecum: Edema, hemorrhage and necrosis.  Cecum: Hemorrhage, moderate.  Cecum: Necrosis, moderate.  Lung: Perivascular eosinophils, minimal. |
| --- | --- | --- | --- |
|  | 33/FD | Lymph Node, Mediastinal: Enlarged, dark, 3x. | Lung: Bacteria, mild.  Lung: Hemorrhage, minimal.  Lung: Inflammation, suppurative, mild.  Lung: Perivascular eosinophils, minimal.  Lymph Node, Mediastinal: Bacteria, Marked.  Lymph Node, Mediastinal: Edema, fibrin, mild.  Lymph Node, Mediastinal: Hemorrhage, minimal.  Lymph Node, Mediastinal: Necrosis/depletion, lymphoid, marked. |
|  | 27/FD | Appendix: Foci, multiple, red, up to 2 x 2  mm. | Appendix: Hemorrhage, mild.  Appendix: Necrosis/depletion, lymphoid, moderate.  Appendix: Infiltration cellular, macrophages, moderate.  Appendix: Note: hemorrhage and necrosis.  Lung: Bacteria, minimal.  Lung: Inflammation, suppurative, minimal.  Lung: Perivascular eosinophils, minimal. |
|  | 31/FD |  | Lung: Bacteria, mild.  Lung: Inflammation, suppurative, minimal. |
|  | 39/FS |  | Lung: Foreign body, minimal.  Lung: Multinucleated giant cells, minimal. |
|  | 21/FS |  | Lung: Unremarkable. |
|  | 38/FS | Lung: Discoloration(s), apical lobe, pale, firm.  Skin: Laceration(s), abdominal, red,  20 x 15 mm. | Lung: Foreign body, moderate.  Lung: Granuloma/pyrogranuloma, moderate.  Lung: Perivascular eosinophils, minimal.  Skin: Inflammation, necrosuppurative, moderate.  Skin: Thrombosis, artery, mild. |

FD = Found Dead, FS = Final Phase Sacrifice, mm = millimeters
