# Supplementary figures and images for: Physiological Responses to Multiple Low-Doses of Bacillus anthracis Spores in the Rabbit Model of Inhalation Anthrax
Source: Pathogens. 2020 Oct 24;9(11):877. doi: 10.3390/pathogens9110877 (PMC7693690; doi:10.3390/pathogens9110877)

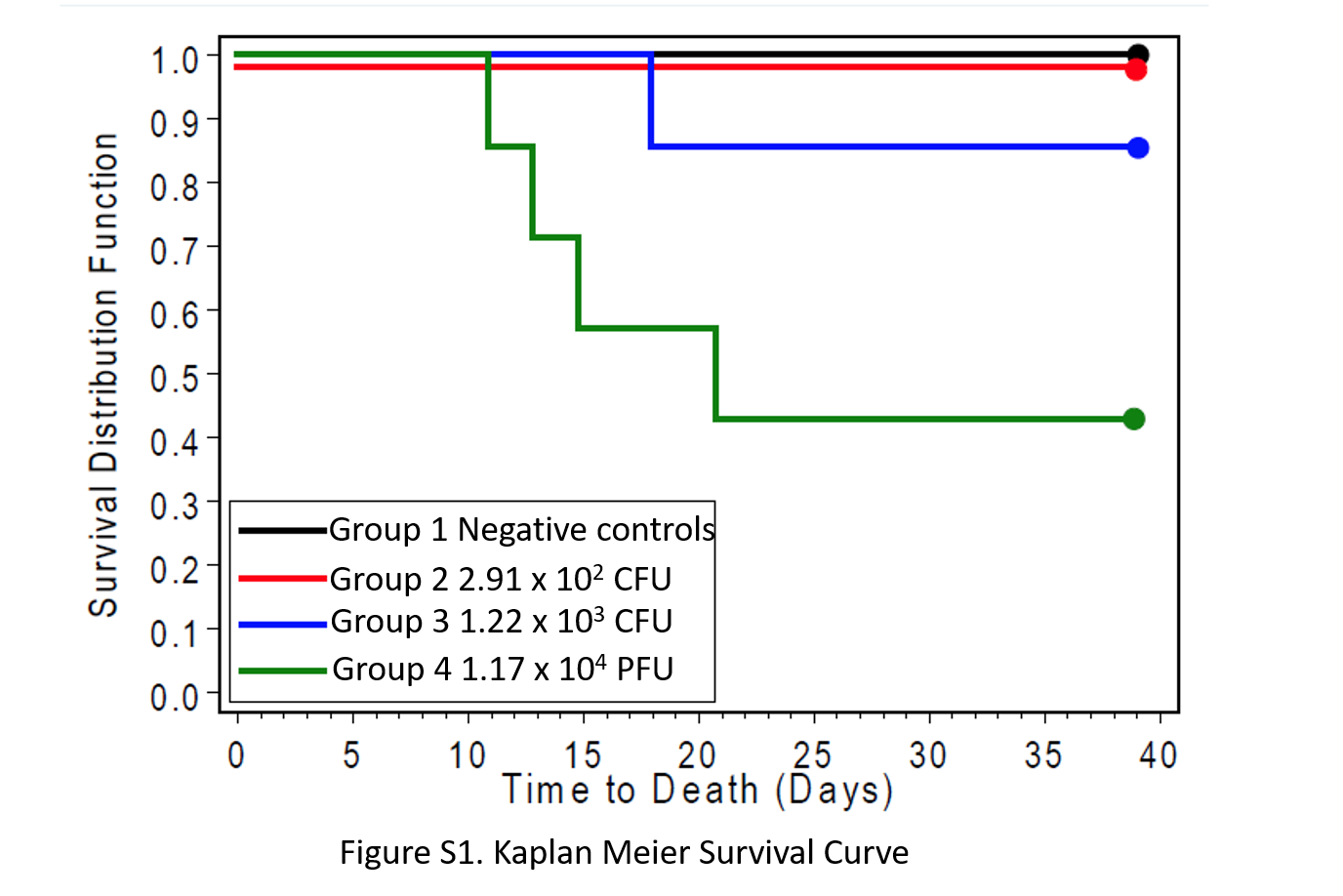

Supplement: Supplementary file 1 [file pathogens-09-00877-s001.zip › figure S1.png]
